# Quantitative approach using multiple single parameters versus visual assessment in dobutamine stress echocardiography

**DOI:** 10.1186/1476-7120-10-31

**Published:** 2012-07-30

**Authors:** Jelena Celutkiene, Diana Zakarkaite, Viktor Skorniakov, Vida Zvironaite, Virginija Grabauskiene, Jelizaveta Burca, Laura Ciparyte, Aleksandras Laucevicius

**Affiliations:** 1 Centre of Innovative Medicine, Zygimantu 9, LT-01102, Vilnius, Lithuania; 2 Clinic of Cardiovascular diseases, Vilnius University Medical Faculty, Santariskiu 2, LT-08661, Vilnius, Lithuania; 3 Faculty of Mathematics and Informatics, Vilnius University, Naugarduko 24, LT-03225, Vilnius, Lithuania

**Keywords:** Coronary artery disease, Coronary stenosis, Dobutamine stress echocardiography, Myocardial deformation imaging, Strain/strain rate imaging, Doppler myocardial imaging, Tissue doppler imaging, Speckle tracking

## Abstract

**Background:**

A number of myocardial Doppler-derived velocity, strain myocardial imaging parameters (DMI) and speckle tracking imaging (STI) have been proposed for the quantification of myocardial ischemia during stress echocardiography. The purpose of the study was to identify the best single ultrasound quantitative parameter for prediction of significant coronary stenosis and compare it with visual assessment during dobutamine stress echocardiography (DSE).

**Methods:**

Prospective analysis included data of 151 patients (age 61.8 ± 9.2) who underwent dobutamine stress echocardiography for known (n = 35) or suspected coronary artery disease (CAD) (n = 36) or symptomatic chest pain (n = 80), excluding patients with previous myocardial infarction. Systolic, post-systolic and diastolic velocities, strain and strain rate parameters were obtained at rest and at peak dobutamine challenge. Derivative markers as E'/A' ratio, post-systolic index and changes from rest to stress were calculated (98 parameters overall, predominantly longitudinal). Coronary angiography was chosen as reference method considering at least one stenosis ≥70% per patient as significant CAD. The predictive value of quantitative parameters and wall motion score index (WMSI) was obtained using logistic regression and ROC analysis.

**Results:**

The value of single parameters discriminated as independent predictors of CAD appeared to be modest (area under the curve [AUC] ranged from 0.63 to 0.72 for 16 PW-DMI, 12 CC-DMI and 12 STI markers), comparing to AUC of WMSI 0.88. Sensitivity, specificity and accuracy of visual DSE evaluation was 82.4% (95%CI 77.4%; 85.2%), 92.6% (95%CI 83.4%; 97.5%) and 86.0% (95%CI 79.5%; 89.6%), respectively, Youden index 0.75. Sensitivity, specificity and accuracy of single predictors ranged from 40.0% to 93.3% (95% CI 22.7%; 99.2%), from 34.2% to 88.7% (95% CI 25.6%; 94.1%) and from 45.8% to 80.0% (95% CI 37.5%; 87.2%) respectively, Youden index ranged from 0.20 to 0.52.

**Conclusions:**

Multiple single quantitative parameters showed limited predictive ability to identify significant coronary artery stenosis. Visual assessment of DSE appears to be more accurate than single velocity and strain/strain rate markers in the diagnosis of CAD.

## Background

Pharmacologic stress echocardiography is an established cost-effective technique for the detection and prognostication of coronary artery disease (CAD) [1], however, it is highly subjective and relies on echocardiographer’s experience. Several novel quantitative technologies have been proposed to overcome this limitation. Assessment of myocardial motion and deformation by means of Doppler based myocardial imaging (DMI), pulsed wave and color coded, as well as Speckle tracking imaging (STI) has been shown to be feasible during dobutamine stress echocardiography (DSE) [2-4].

A number of myocardial velocity and strain parameters derived by DMI and STI are reported to be reliable quantitative markers of stress-induced ischemia. They include reduced response of systolic and diastolic velocities, prominent post-systolic velocities [5-8], reduced strain and strain rate, increased ratio of post-systolic shortening to maximal segmental deformation, delayed relaxation measured by transverse strain at peak stress [9-13]. However, the translation of these tools into routine clinical practice raises the question of which is the most accurate and the most reliable parameter. Moreover, no data to date show a better and higher performance of these tools when compared to conventional wall motion analysis, and they are not recommended by the European Association of Echocardiography stress echocardiography expert consensus statement [14].

This case–control study aimed to discriminate the best single quantitative parameter for prediction of significant coronary stenosis from the wide range of velocity- and strain-based parameters, simultaneously applying pulsed wave DMI (PW-DMI), color coded DMI (CC-DMI) and STI during dobutamine stress echocardiography (DSE) test and compare them with conventional visual wall motion assessment.

## Methods

Prospective analysis included data of 151 patients (age 61.8 ± 9.2) who underwent dobutamine stress echocardiography for known (n = 35) or suspected CAD (n = 36) or symptomatic chest pain (n = 80). Typical angina was found in 63 (41.7%) patients, while remaining patients presented atypical angina or other ischemic equivalents. Exercise ECG could not be performed or did not provide definite results in 105 (69.6%) patients. In order to shape homogenous study group in terms of baseline quantitative parameters patients with previous myocardial infarction were excluded. Previous myocardial infarction was identified either from medical history or from revealing the combination of resting wall motion abnormalities with silent corresponding coronary occlusion. Other exclusion criteria were previous cardiac surgery, permanent pacemaker, nonsinus rhythm, significant valvular heart disease, significant left ventricular hypertrophy (myocardial mass index >120 g/m^2^), atrial or ventricular arrhythmias, bundle branch blocks or LV EF <45%. Mean wall motion score index (WMSI) at rest was equal to 1.02 ± 0.04 and mean left ventricular (LV) ejection fraction (EF) equal to 54.5 ± 1.8%. The study protocol was approved by the local ethics committee.

### Dobutamine echocardiography and visual assessment

Each study patient underwent DSE. Betablocking medications were discontinued 48 hours prior to the study. Dobutamine was infused at 5, 10, 20, 30, and 40 *μ*g/kg/min for 3 minutes each stage. Atropine up to 1 mg was added if necessary. The conventional echocardiograph (System Vivid 7, GE Healthcare, *Horten, Norway*) with 1,5 – 4,6 MHz transducer was used. A 12-lead electrocardiogram (ECG), blood pressure (BP), and standard two-dimensional echocardiograms were taken at baseline, low-dose, peak dobutamine levels and during recovery. The dobutamine infusion was terminated once 85% of the maximal predicted heart rate was achieved. Stress test was terminated prematurely in the presence of severe chest pain or other intolerable symptoms, severe arrhythmia, >2 mm ST-segment elevation or depression, systolic blood pressure >230 mm Hg, diastolic blood pressure >120 mm Hg, or a drop in systolic blood pressure >20 mm Hg.

The long and short axis of the left ventricle from parasternal window, 4- and 2-chamber views from apical window were acquired for comparison in four stages of stress test. Left ventricle was divided into 16 myocardial segments according to the recommendations of the American Society of Echocardiography for the assessment of local myocardial function by wall thickening and endocardial motion. Each myocardial segment was scored as 1 if normokinetic, 2 if hypokinetic, 3 if akinetic and 4 if dyskinetic, assessment was made using quad-screen display by experienced observers. It was considered that in some cases of normal variant basal inferior and basal inferoseptal segments could look and be scored as hypokinetic. The sum of all segmental scores divided by the number of assessed segments made wall motion score index.

### Acquirement of quantitative parameters

PW-DMI profiles, CC-DMI and STI images were recorded at baseline and peak dobutamine levels with breath-holding. Images were optimised for pulse repetition frequency, color saturation, sector size, and depth to allow high frame rates. The loops were stored digitally on hard disc and analysed off-line using customised software (Echopac PCBT08, GE Healthcare).

### Pulsed-wave doppler myocardial imaging

Apical 2, 3 and 4-chamber views were used for the acquirement of longitudinal myocardial velocity profiles by pulsed-wave DMI. The frequency of transducer in pulsed-wave Doppler mode was 2.6 MHz. A sampling gate of 6.4 mm was placed at the centre of each evaluated segment. The spectral Doppler signal parameters were adjusted to obtain Nyquist limit 40 cm/s by using the lowest filter settings and the optimal gain to minimize noise. Special care was taken to align the ultrasound beam parallel to the direction of motion of each myocardial segment (the angle between the scan line and the myocardial segment did not exceed 30°) and to avoid lateral displacement of the sample volume outside the segment.

### Color coded doppler myocardial imaging

The frame rate of stored apical 2, 3 and 4-chamber cineloops was in the range of 140–150 frames/sec. A sample cursor was placed manually at the midpoint of each evaluated segment in the 3 apical views, and myocardial velocity, strain and strain rate curves were reconstituted off-line.

### Speckle tracking myocardial imaging

The frame rate of stored apical 2, 3 and 4-chamber cineloops for speckle tracking analysis was in the range of 70–90 frames/sec. After manual tracing of endocardial borders in the end-systolic frame of the 2-D images, the software automatically tracked myocardial motion, creating 6 regions of interest in each apical image, with tracking quality labeled as verified or unacceptable. In segments with unacceptable tracking, the observer readjusted the endocardial trace line until a verified tracking was achieved. If this was not attainable, that segment was excluded from analysis. Graphical displays of deformation parameters (reflecting the average value of all of the acoustic markers in each segment) were then automatically generated for 6 segments in each view.

### Measurement of quantitative parameters

**Figure 1:**
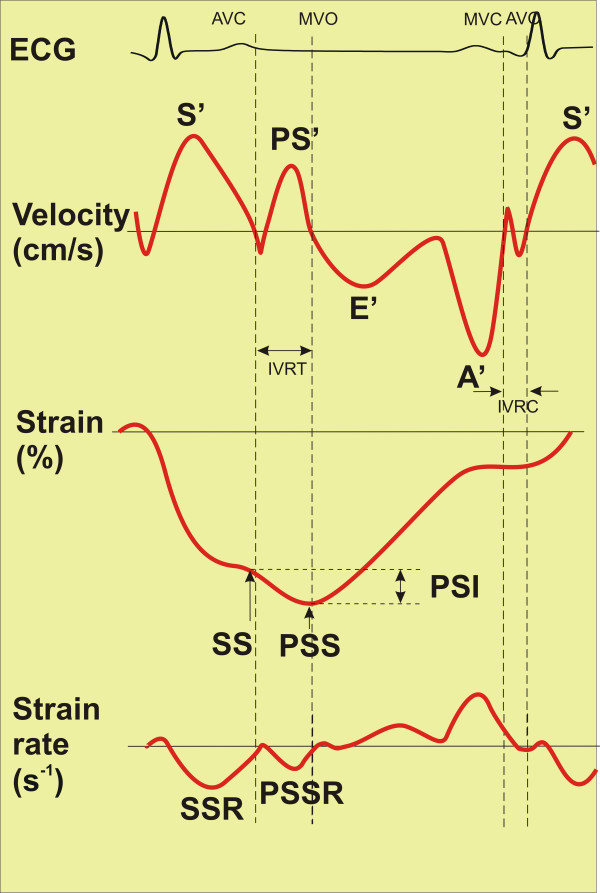
**Schematic of measured study parameters.** At the top velocity markers are presented, in the middle – strain markers, at the bottom – strain rate markers. S' = peak systolic velocity, PS' = peak post-systoloc velocity, E' = early diastolic wave, A' = late diastolic wave, SS = peak systolic strain, PSS = peak post-systolic strain, PSI = post-systolic index, SSR = systolic strain rate, PSSR = post-systolic strain rate, AVC = aortic valve closure, MVO = mitral valve opening, MVC = mitral valve closure, AVO = aortic valve opening, IVRT = isovolumic relaxation time, IVCT = isovolumic contraction time.

Peak longitudinal systolic (S'), post-systolic (PS'), diastolic (E', A') velocities, peak systolic (SS) and post-systolic (PSS) strain, peak systolic (SSR) and post-systolic (PSSR) strain rate at rest and during peak stress were measured (Figure 1). Besides longitudinal markers, peak radial systolic (RSSR) and post-systolic strain rate (RPSSR) were measured using STI method. Absolute and relative differences of the paramteters between rest and stress, as well as E'/A' ratio, post-systolic index, ratio of post-systolic index to systolic and post-systolic strain were calculated. Full list of 98 measured and calculated parameters is presented in Additional file 1.

Cardiac cycles associated with atrial or ventricular extrasystolic beats were excluded. Quantitative parameters were analyzed by investigators who were blinded to the coronary angiography findings. All the measurements were performed manually off-line. Five consecutive beats were averaged for each of these measurements to improve the signal to noise ratio of the derived curves.

Event timing was performed using PW-DMI velocity profile of basal inferoseptal or basal inferior segment. The duration of positive systolic velocity wave was taken as systole. Post-systolic motion in PW-DMI mode was defined as positive wave, which appeared after the curve of systolic ejection had reached the zero line (Figure 1, top). At high heart rate the time point of zero crossing after the initial negative velocity after ejection in velocity/time curves served as a marker for aortic valve closure [15].

Post-systolic strain (Figure 1, middle) was defined as a continuation of negative strain wave after the aortic valve closure till the peak of the wave or the second negative peak occuring in the period of isovolumic relaxation (IVRT) up to 50–70 ms after the mitral valve opening. Post-systolic index (PSI) was defined as subtraction:

### *Post-systolic index = post-systolic strain – systolic strain*

Peak longitudinal systolic strain rate was defined as maximal negative peak in strain rate curve during systole, post-systolic longitudinal systolic strain rate was measured as negative peak during IVRT up to 50–70 ms after the mitral valve opening (Figure 1, bottom). Radial strain rate is obtained from apical window by STI method being positive in normal setting. Time to peak systolic velocity and peak post-systolic strain was measured from the onset of QRS complex.

All quantitative parameters were evaluated in 9 out of 16 myocardial segments, discriminated according to our previous DMI study [5] as most representative for three coronary territories. Basal inferoseptal, basal inferior and mid inferior segments were attributed to right coronary artery (RCA); mid inferoseptal, apical inferoseptal, basal anterior and mid anteroseptal segments were attributed to left anterior descending (LAD); basal inferolateral and mid inferolateral segments were attributed to left circumflex artery (LCX). Additionally to this prevalent distribution myocardial segments were correlated to the circulation supply according to the individual type of the coronary circulation in each patient [16].

The STENOSED group consisted of segments supplied by stenosed (≥70% narrowing) coronary arteries, while the NON-STENOSED group consisted of segments supplied by non-stenosed (normal or stenosis <70%) coronary arteries (total number of segments 1359).

Ejection fraction was calculated using the modified biplane Simpson method [17]. LV myocardial mass index (MMI) was calculated by the Devereux formula [17].

### Interobserver and intraobserver agreement

Interobserver agreement was determined by having two independent investigators measure representative parameters with three quantitative methods and assess WMSI in 15 randomly selected patients. Intraobserver agreement was determined by having 1 investigator repeat measurements and WMSI evaluation in other 15 randomly selected patients 1 month later, while being blinded to the previous measurements. Reproducibility is expressed as the mean percentage difference (value of observer 1 - value of observer 2/mean of the values of observer 1 and 2).

### Coronary angiography

The coronary angiography was performed in all 151 patients referred to DSE within 6–8 weeks after dobutamine challenge in Vilnius University Hospital Santariskes Clinic using a standard technique with *Inova2100* (*GE Healthcare*). Clinical decision to perform coronary angiography was made independently of this study by consulting cardiologists, who frequently were aware of the results of DSE visual evaluation. Coronary angiographic data were analysed visually by 2 experts blinded to the clinical data and the results of DSE. Significant coronary stenosis has been defined as ≥70% artery lumen narrowing localized in the proximal or middle segments of the coronary arterial tree in 2 orthogonal angiograms.

### Statistical analysis

Descriptives were presented as mean+/−standard deviation. Comparison of STENOSED versus NON-STENOSED segments with respect to different markers of interest was carried out with a help of *t*-test (for independent samples) or Mann–Whitney test. As the amplitude of quantitative parameters depends on the location of the segment in the left ventricle, analysis was performed separately for each segment location. The level of significance was set to 0.05. All reported *P* values were two sided.

The evaluation of predictive ability of different imaging methods was carried out using logistic regression and receiver operating curve (ROC) analysis. In each segment, markers which had predictive ability (area under ROC significantly differed from 0.5, level of significance 0.025) were discriminated. To build logistic regression model SPSS 16.0 software was used. Sensitivity, specificity and accuracy were calculated using standard definitions; cut-offs were chosen as having the highest Youden index, which was calculated as [1- (1 - sensitivity) + (1 - specificity)] [1]. In order to compare the predictive value of elicited parameters comparisons of areas under receiver operating curves (AUC) were made using method described elsewhere [18].

## Results

### Stress echocardiography

Clinical and echocardiographic characteristics of the study population are reported in Table 1. No major complications occurred during DSE. The 85% age-predicted maximum heart rate was achieved in 137 (90.7%) patients. Other reasons for premature termination of dobutamine challenge were as follows: chest pain in 8 (5.3%) patients, prominent wall motion abnormalities (1 patient), hypotension (2 patients), intolerable symptoms (1 patient) and sustained ventricular arrhythmia (2 patients). Hemodynamic features of the tests are presented in Table 1.

**Table 1:** Baseline characteristics of study group and dobutamine tests hemodynamics

**Characteristics**	**Study group (n = 151)**
Age, years	61.77 ± 9.21
Male	89 (58.9 %)
Typical angina	63 (41.7 %)
Exercise ECG not performed	59 (39.1 %)
Exercise ECG negative	16 (10.6 %)
Exercise ECG positive	30 (19.9 %)
Exercise ECG non-diagnostic	46 (30.5 %)
Hypertension	141 (93.4 %)
Hypercholesterolemia	118 (78.1 %)
Diabetes	29 (19.2 %)
Overweight	101 (66.9 %)
Smoking	28 (18.5 %)
Family history of CAD	39 (25.8 %)
Beta-blockers	96 (63.6 %)
Ca channel blockers	56 (37.1 %)
ACEI/ARB	105 (69.5 %)
Nitrates	38 (25.2 %)
Diuretics	40 (26.5 %)
Aspirin	71 (47.0 %)
MMI, g/m^2^	99.42 ± 17.13
EF rest, %	54.49 ± 1.76
EF stress, %	59.86 ± 6.63
HR rest, min^-1^	69.88 ± 11.13
HR stress, min^-1^	132.43 ± 10.92
Systolic BP rest, mm Hg	139.89 ± 30.44
Systolic BP stress, mm Hg	144.22 ± 26.13
Diastolic BP rest, mm Hg	81.96 ± 11.44
Diastolic BP stress, mm Hg	71.38 ± 13.72
ECG changes during stress	77 (51.0 %)
Chest pain during stress	87 (57.6 %)
WMSI rest	1.02 ± 0.04
WMSI stress	1.21 ± 0.23

ECG = Electrocardiogram; CAD = coronary artery disease; MMI = myocardial mass index; EF = ejection fraction; HR = heart rate; BP = blood pressure; WMSI = wall motion score index; ACEI = angiotensin converting enzyme inhibitors; ARB = angiotensin receptor blockers.

Ischemia was visually assessed in 60 (39.7%) subjects: rest and peak WMSI was 1.02 ± 0.05 and 1.36 ± 0.21, respectively. A normal test was detected in 91 individuals (60.3%).

### Angiographic results

A total of 53 (35.1%) patients had ≥70% coronary stenosis and 98 had normal coronary anatomy or <70% stenosis. Among the patients with significant coronary stenosis, 1-vessel disease was present in 32, 2-vessel disease in 11, and 3-vessel disease in other 11 patients. There was significant LAD stenosis in 34, LCX stenosis in 24, and RCA stenosis in 28 patients. The grade of stenosis ≥70% was found in 48 arteries, ≥90% – in 38 arteries. The majority of patients (122) had right dominance, 21 patients presented left dominance, and 8 patients had balanced type of coronary circulation.

### Feasibility and reproducibility of quantitative data

The stored images of 38 segments (2.8%) due to poor image quality were totally excluded from the analysis, therefore the data of 1321 (97.2%) segments were finally analysed. The prevalence of uninterpretable signals in the segments included in the final analysis for PW-DMI, CC-DMI and STI at rest was 1.8%, 0.6% and 2.1%, during stress 3.8%, 3.3% and 5% and in total 2.8%, 1.9% and 3.6%, respectively. One of the most frequently (15-20% of segments) unmeasurable marker was stress E' wave velocity due to the E' and A' waves fusion at higher heart rates, especially with CC-DMI and STI methods. The best inter- and intraobserver reproducibility is documented for strain rate, while strain parameters appeared to be more variable with both CC-DMI and STI. The mean percentage differences of inter- and intraobserver measurements of velocity, strain, strain rate by three quantitative methods and WMSI are summarized in Table 2.

**Table 2:** Reproducibility of visual and three quantitative methods (mean percentage difference)

	**Visual**	**PW-DMI**	**CC-DMI**			**STI**		
	***WMSI***	***velocities***	***velocities***	***strain***	***strain rate***	***velocities***	***strain***	***strain rate***
***Interobserver***	0.035	0.059	0.038	0.036	0.007	0.031	0.077	0.009
***Intraobserver***	0.028	0.091	0.004	0.116	0.016	0.037	0.109	0.016

PW-DMI = pulsed wave Doppler myocardial imaging; CC-DMI = color coded Doppler myocardial imaging; STI = speckle tracking imaging; WMSI = wall motion score index.

### Single parameters - predictors of stenosis

The most informative single parameters for prediction of significant coronary stenosis discriminated by means of ROC analysis are presented in Table 3. They comprised 16 PW-DMI, 12 CC-DMI and 12 STI markers with modest value of AUC from 0.63 to 0.72. They were 23 velocity, 10 strain and 7 strain rate markers specific for each segment location. Among stenosis predictors there were 9 rest parameters, 19 stress parameters and absolute or relative changes of 12 markers from rest to stress. Mean values of quantitative parameters, discriminated as significant predictors of stenosis, in STENOSED and NON-STENOSED myocardial segments are presented in Additional file 2.

**Table 3:** Single parameters selected as significant predictors of coronary stenosis in separate myocardial segments

**Method**	**Parameter**	**Cut-off**	**AUC (95 % CI)**	**Sensitivity % (95 % CI)**	**Specificity % (95 % CI)**	**Accuracy % (95 % CI)**	**Youden index**
***Basal inferoseptal segment***							
PW-DMI	T to S'_*stress*_	70.5	0.66* (0.54;0.78)	56.7 (37.4;74.5)	76.7 (68.1;83.9)	72.7 (64.8;79.6)	0.33
	S'_*stress*_*-* S'_*rest*_	4.15	0.65* (0.53;0.76)	56.7 (37.4;74.5)	73.1 (64.2;80.8)	69.8 (61.7;77.0)	0.30
	A'_*stress*_	12.3	0.64* (0.53;0.75)	46.7 (28.3;65.7)	81.4 (73.0;88.1)	74.1 (66.1;81.1)	0.28
CC-DMI	E'/A'_*rest*_	0.64	0.66* (0.55;0.76)	64.3 (44.1;81.4)	68.2 (58.6;76.7)	67.4 (58.9;75.1)	0.33
STI	E'_*stress*_	4.5	0.70* (0.58;0.83)	66.7 (44.7;84.4)	71.6 (61.0;80.7)	70.5 (61.2;78.8)	0.38
	E'/A'_*stress*_	0.34	0.66* (0.52;0.80)	47.6 (25.7;70.2)	88.1 (79.2;94.1)	80.0 (71.1;87.2)	0.36
***Mid inferoseptal segment***							
CC-DMI	PSI_*stress*_/SS_*stress*_	0.13	0.65* (0.54;0.77)	58.1 (39.1;75.5)	69.1 (59.6;77.6)	66.7 (58.2;74.4)	0.27
	SSR_*stress*_ - SSR_*rest*_	−0.21	0.64* (0.53;0.74)	74.2 (55.4;88.1)	55.9 (46.1;65.3)	59.9 (51.3;68.0)	0.30
***Apical inferoseptal segment***							
STI	PSI_*rest*_/SS_*rest*_	−0.15	0.65*(0.54;0.76)	70.0 (50.6;85.3)	57.9 (48.3;67.1)	60.4 (51.9;68.5)	0.28
	PSI_*rest*_	3.85	0.65*(0.54;0.76)	70.0 (50.6;85.3)	56.1 (46.5;65.4)	59.0 (50.5;67.1)	0.26
	RSSR_*rest*_	1.95	0.63*(0.53;0.74)	90.0 (73.5;97.9)	34.2 (25.6;43.7)	45.8 (37.5;54.3)	0.24
***Basal inferior segment***							
PW-DMI	S'_*stress*_	10.8	0.65* (0.53;0.76)	48.3 (29.4;67.5)	77.3 (68.7;84.5)	71.6 (63.6;78.7)	0.26
	S'_*stress*_*-* S'_*rest*_	4.8	0.64* (0.53;0.75)	75.9 (56.5;89.7)	52.1 (42.8;61.3)	56.8 (48.4;64.9)	0.28
	T to S'_*stress*_ - T to S'_*rest*_	−45.5	0.64* (0.53;0.75)	62.1 (42.3;79.3)	65.5 (56.3;74.0)	64.9 (56.6;72.5)	0.28
	(T to S'_*stress*_ - T to S'_*rest*_)/T to S'_*rest*_	−0.44	0.66* (0.55;0.76)	69.0 (49.2;84.7)	58.0 (48.6;67.0)	60.1 (51.8;68.1)	0.27
	E'_*rest*_	9.35	0.64* (0.54;0.74)	93.3 (77.9;99.2)	34.2 (25.8;43.4)	46.0 (37.8;54.3)	0.28
	E'_*stress*_	7.53	0.72* (0.62;0.82)	72.4 (52.8;87.3)	66.1 (56.7;74.7)	67.4 (59.1;74.9)	0.39
CC-DMI	PSI_*stress*_/PSS_*stress*_	0.06	0.65* (0.52;0.78)	66.7 (44.7;84.4)	61.9 (51.4;71.5)	62.8 (53.6;71.4)	0.29
STI	SS_*rest*_	−13.0	0.65* (0.54;0.77)	41.4 (23.5;61.1)	88.7 (81.4;93.8)	79.2 (71.6;85.5)	0.30
***Mid inferior segment***							
PW-DMI	T to S'_*stress*_ - T to S'_*rest*_	−41.9	0.64* (0.53;0.75)	66.7 (47.2;82.7)	64.1 (54.7;72.8)	64.6 (56.3;72.3)	0.31
CC-DMI	SS_*stress*_	−14.5	0.66* (0.56;0.76)	75.0 (55.1;89.3)	55.8 (46.1;65.1)	59.6 (51.0;67.7)	0.31
STI	PSSR_*rest*_	−0.65	0.64* (0.52;0.76)	41.4 (23.5;61.1)	87.0 (79.4;92.5)	77.8 (70.1;84.3)	0.28
	(PSI_*stress*_ - PSI_*rest*_)/PSI_*rest*_	−0.08	0.67* (0.56;0.78)	48.3 (29.4;67.5)	81.3 (72.8;88.0)	74.5 (66.4;81.4)	0.30
***Basal anterior segment***							
PW-DMI	S'_*stress*_	8.2	0.63* (0.52;0.74)	75.8 (57.7;88.9)	44.2 (34.9;53.9)	51.4 (43.0;59.7)	0.20
CC-DMI	S'_*stress*_	6.2	0.69* (0.59;0.79)	51.7 (32.5;70.6)	81.3 (72.8;88.0)	75.2 (67.2;82.1)	0.33
	(PS'_*stress*_ - PS'_*rest*_)/PS'_*rest*_	3.1	0.69* (0.54;0.84)	53.3 (26.6;78.7)	83.1 (71.0;91.6)	77.0 (65.8;86.0)	0.36
	SSR_*stress*_	−2.4	0.65* (0.54;0.75)	80.0 (61.4;92.3)	52.7 (43.0;62.2)	58.5 (49.9;66.7)	0.33
STI	PSI_*stress*_ - PSI_*rest*_	−5.5	0.64* (0.53;0.75)	40.0 (22.7;59.4)	87.3 (79.6;92.9)	77.1 (69.3;83.8)	0.27
	PSSR_*rest*_	−1.1	0.68* (0.58;0.78)	80.0 (61.4;92.3)	47.4 (37.9;56.9)	54.2 (45.7;62.5)	0.27
	PSSR_*stress*_ - PSSR_*rest*_	−0.39	0.67* (0.56;0.78)	80.0 (61.4;92.3)	56.8 (47.0;66.1)	61.7 (53.1;69.8)	0.37
***Basal inferolateral segment***							
PW-DMI	S'_*stress*_	12.1	0.65* (0.54;0.76)	70.4 (49.8;86.2)	57.3 (47.8;66.4)	59.7 (51.2;67.8)	0.28
	E'_*stress*_	8.1	0.66* (0.53;0.78)	64.0 (42.5;82.0)	68.7 (59.4;77.0)	67.9 (59.4;75.5)	0.33
	E'/A'_*stress*_	0.51	0.69* (0.57;0.80)	56.0 (34.9;75.6)	76.3 (67.4;83.8)	72.7 (64.5;79.9)	0.32
***Mid inferolateral segment***							
CC-DMI	E'/A'_*rest*_	0.5	0.65* (0.54;0.76)	61.5 (40.6;79.8)	70.9 (61.5;79.2)	69.1 (60.6;76.8)	0.32
	SS_*stress*_	−16.0	0.72* (0.62;0.82)	80.8 (60.6;93.4)	59.8 (50.1;69.0)	63.8 (55.2;71.8)	0.41
	SSR_*stress*_	−1.4	0.68* (0.56;0.80)	64.0 (42.5;82.0)	71.2 (61.8;79.4)	69.9 (61.4;77.4)	0.35
***Mid anteroseptal segment***							
PW-DMI	PS'_*stress*_ - PS'_*rest*_	1.8	0.66* (0.55;0.76)	86.2 (68.3;96.1)	52.3 (42.5;62.1)	59.6 (50.8;67.9)	0.39
	(PS'_*stress*_ - PS'_*rest*_)/PS'_*rest*_	0.86	0.70* (0.58;0.82)	81.8 (59.7;94.8)	70.2 (59.3;79.7)	72.6 (63.1;80.9)	0.52
CC-DMI	E'_*stress*_	3.5	0.72* (0.61;0.83)	84.6 (65.1;95.6)	51.2 (39.9;62.4)	59.3 (49.4;68.6)	0.36
STI	PSI_*stress*_/SS_*stress*_	0.14	0.64* (0.54;0.75)	67.9 (47.6;84.1)	59.0 (49.0;68.5)	60.9 (52.1;69.2)	0.27

*P* ≤ 0.025 for AUC > 0.5, * *P* < 0.05 compared with AUC of visual assessment 0.88; PW-DMI = pulsed wave Doppler myocardial imaging; CC-DMI = color coded Doppler myocardial imaging; STI = speckle tracking imaging; AUC = area under the curve; CI = confidence interval; S' = peak systolic velocity; PS' = peak post-systoloc velocity; E' = early diastolic wave; A' = late diastolic wave; SS = peak systolic strain, PSS = peak post-systolic strain; PSI = post-systolic index; SSR = systolic strain rate; PSSR = post-systolic strain rate; T = time; RSSR = radial systolic strain rate; RPSSR = radial post-systolic strain rate.

According to the size of AUC the strongest single predictors of stenosis appeared to be stress E' wave velocity (PW-DMI, basal inferior segment, cut-off 7.53 cm/s, and CC-DMI, mid anteroseptal segment, cut-off 3.5 cm/s) and stress systolic strain (CC-DMI, mid inferolateral segment, cut-off −16%).

### Diagnostic accuracy of quantitative parameters and wall motion analysis

Sensitivity, specificity and accuracy of single predictors ranged from 40.0% to 93.3% (95% CI 22.7%; 99.2%), from 34.2% to 88.7% (95% CI 25.6%; 94.1%) and from 45.8% to 80.0% (95% CI 37.5%; 87.2%), respectively, Youden index ranged from 0.20 to 0.52 (see Table 3). Meanwhile, sensitivity, specificity and accuracy of visual DSE evaluation was 82.4% (95% CI 77.4%; 85.2%), 92.6% (95% CI 83.4%; 97.5%) and 86.0% (95% CI 79.5%; 89.6%), respectively, Youden index 0.75. Comparing AUCs of WMSI (0.88) with each quantitative marker it was found, that visual DSE evaluation was significantly better than all discriminated quantitative predictors (P < 0.05).

## Discussion

The present study shows that in patients with suspected CAD undergoing dobutamine stress echocardiography quantitative tools are not superior to visual wall motion analysis. None of the single parameters investigated was able to identify myocardial ischemia and significant coronary artery disease with a comparable diagnostic accuracy vs. wall motion analysis. The several parameters did not perform differently between themselves. Moreover, these tools are quite time consuming and do not seem to offer a significant diagnostic improvement in the hands of expert echocardiographers.

### Comparison with previous studies

There are not many studies comparing multiple quantitative parameters during stress echocardiography. This approach aimed to identify the best quantitative parameter for the diagnosis of myocardial ischemia and significant coronary artery disease. The rationale of applying pulsed wave and color coded Doppler myocardial imaging as well as speckle tracking technique during the same dobutamine stress test is based on the awareness that each of these methods has inherent advantages and limitations. Direct comparison of physiologically different parameters as well as similar markers obtained by different methods should allow the reasonable choice of the most reliable predictor of CAD for practical use. In this, our approach is unique and provides a missing piece of information.

We performed the search of the best predicting parameter of significant stenosis separately for each evaluated myocardial segment, taking into account known base-to-apex and wall-to-wall differencies of myocardial velocity and strain/strain rate [11,19-21]. From the list of 98 rest and stress measured and calculated parameters, 40 site-specific markers appeared to be statistically significant predictors of coronary stenosis (AUC >0.5, *P*≤0.025). Thus, our results confirm the relation of substantial list of quantitative parameters of regional myocardial function to the obstruction of coronary arteries.

In agreement with previously published findings [6,8,11,22,23] blunted response of systolic velocity to dobutamine infusion and prolonged time to peak systolic velocity during stress demonstrated by Doppler based methods appeared to be significant predictors of coronary stenosis.

Also, in concordance with previous reports [7,24-26] all quantitative techniques employed in the present study provided markers of regional diastolic dysfunction as significant predictors of stenosis. E' wave velocity and E'/A' ratio during stress demonstrated the predictive ability in all coronary territories. Furthermore, we found significant deterioration of local diastolic function in the segments supplied by stenosed RCA even at rest.

In parallel to previous deformation studies [11-13] longitudinal myocardial deformation was significantly impaired during stress in STENOSED segments. Stress systolic strain, ratio of post-systolic index to systolic and post-systolic strain, absolute and relative changes of post-systolic index appeared to be significant predictors of CAD. Besides, systolic strain and post-systolic index were significantly lower in STENOSED segments already at baseline.

By analogy with the prior clinical studies [11,13,27], longitudinal as well as radial strain rate markers at rest and during stress appeared to be significant predictors of CAD in our data. Stress markers included longitudinal systolic and post-systolic strain rate, absolute changes in longitudinal systolic and post-systolic strain rate. Rest markers included longitudinal post-systolic and radial systolic strain rate in the segments supplied by stenosed RCA and LAD.

However, the predictive ability of discriminated single parameters appeared to be modest: AUC ranged from 0.63 to 0.72. Similar ability to predict significant CAD was reported for strain rate, strain parameters and post-systolic index (AUCs 0.67 – 0.71, 0.64 - 0.66, and 0.60 – 0.63, respectively) in the study of Hanekom et al. [4]. Remarkably, the majority of informative parameters in the present study were not repetitive from segment to segment, and in the same segment different imaging methods provided various markers. Thereby, analysing the extensive set of quantitative indices we could not distinguish any single robust predictor of coronary stenosis universal for the majority of myocardial segments.

None of the quantitative markers could compare with the visual assessment of DSE in terms of the accuracy of predicting stenosis, which was 86% in the present study (accuracy of single parameters ranged from from 45.8% to 80.0%). In MYDISE investigation similar limited sensitivity (67-69%) and specificity (60-67%) of myocardial systolic velocities before correction by logistic regression models were demonstrated [22]. In the study of Cain et al. [28] the accuracy of myocardial Doppler velocities was lower comparing to wall motion scoring, while strain rate imaging in the report of Voigt et al. [11] was found to be comparable with conventional visual assessment. Investigating the accuracy of Doppler-based and two-dimensional strain imaging, Hanekom [4] did not find significant differences between quantitative and visual assessment.

Limited value of distinguished indices largely could be attributed to known technical challenges of quantitative imaging: potentially inadequate spatial and temporal resolution, angle-dependency, less accurate tracking of ultrasound speckles at higher heart rates, noise and artefacts [29]. Mutual interaction of STENOSED and NON-STENOSED segments, possibly, may diminish the differences between local myocardial motion markers of these two groups [30]. Though strain/strain rate parameters were supposed to overcome the influence of adjacent segments, our data demonstrate interfering greater variability of strain markers.

Current lack of evidence on effective application of quantitative methods in routine practice is reflected in recommendation documents of EAE and ASE [14,31]. These new methods are not routinely recommended for detection of myocardial ischemia in stress echocardiography. Recent consensus statement [32] also claims that in the majority of areas, including assessment of ischemic myocardium and stress echocardiography, quantitative methodology is not yet ready for routine clinical use.

### Study limitations

We are aware that selected angiographic coronary stenosis does not always reflect the potential alteration in the regional myocardial perfusion. Although coronary angiography is widely accepted as the reference standard, the relationship between stenosis severity and physiological reduction of coronary flow is quite variable. Qualitative assessment of coronary stenosis, though reflects common clinical practice, constitutes another limitation of the study. Further research in creating user-friendly automated quantitative tool is warranted.

## Conclusions

Though stress echocardiography is a well established tool for the detection of coronary artery disease, visual analysis of regional myocardial function has a drawback of substantial subjectivity and inter-observer variability. Therefore, the implementation of an objective, operator-independent technique using novel quantitative methods seems to be an attractive clinical goal but still remains the Holy Grail in echocardiography.

We failed to find single powerful quantitaive parameter applicable in each myocardial segment for prediction of coronary stenosis. Visual assessment appears to be more accurate than single velocity and strain/strain rate markers in the diagnosis of coronary artery disease. The need still remains to create more practical and less time-consuming quantitative tool for DSE interpretation.

## Abbreviations

DMI: doppler myocardial imaging; STI: speckle tracking imaging; PW-DMI: pulsed wave doppler myocardial imaging; CC-DMI: color coded doppler myocardial imaging; DSE: dobutamine stress echocardiography; LV EF: left ventricular ejection fraction; ROC: receiver operating curve; AUC: area under the curve; ECG: electrocardiogram; BP: blood pressure; S': systolic velocity; PS': post-systolic velocity, systolic; E', early diastolic velocity; A', late diastolic velocity; SS, systolic strain; PSS: post-systolic strain; SSR: systolic strain rate; PSSR: post-systolic strain rate; RSSR: radial systolic strain rate; RPSSR: radial post-systolic strain rate; IVRT: isovolumic relaxation time; PSI: post-systolic index; RCA: right coronary artery; LAD: left anterior descending artery; LCX: left circumflex artery; MMI: myocardial mass index; CAD: coronary artery disease.

## Competing interests

The authors declare that they have no competing interests.

## Authors’ contribution

JC participated in design creation, performed quantitative imaging during dobutamine stress tests, measured study parameters, participated in data analysis and drafting article. DZ participated in concept formulation and drafting article. VS performed statistical data analysis, helped to interpret the data and draft article. VZ participated in design creation and drafting article, performed critical revision of article. VG participated in data analysis, performed critical revision of article. JB collected and measured study parameters, participated in data analysis. LC collected and measured study parameters, participated in data analysis. AL participated in design creation and drafting article, performed critical revision of article. All authors read and approved the final manuscript.

## Authors’ information

JC since 1999 works and since 2006 runs the unit of stress echocardiography tests, where about 1500 tests per year are performed.

## Supplementary Material

Additional file 1 **Celutkiene_ additional file 1.doc: Additional file 1.** Full list of measured and calculated parameters. Click here for file

Additional file 2 **Celutkiene additional file 2 70.doc: Additional file 2.** Mean values of significant predictors of stenosis in NON-STENOSED and STENOSED segments. Click here for file
