# Supplementary material for: Quantitative approach using multiple single parameters versus visual assessment in dobutamine stress echocardiography
Source: Cardiovasc Ultrasound. 2012 Jul 30;10:31. doi: 10.1186/1476-7120-10-31 (PMC3495225; doi:10.1186/1476-7120-10-31)
Supplement: Additional file 1 — Celutkiene_ additional file 1.doc: Additional file 1. Full list of measured and calculated parameters. [file 1476-7120-10-31-S1.pdf]

Additional file 1. Full list of measured and calculated parameters

Nr	Method	Parameter	Abbreviation	Dimension
1	PW-DMI: longitudinal axis	Peak systolic velocity Rest	S'_{rest}	cm/s
2		Peak systolic velocity Stress	S'_{stress}	cm/s
3		Peak systolic velocity (Stress - Rest)	S'_{stress} - S'_{rest}	cm/s
4		Peak systolic velocity [(Stress - Rest)/Rest]	(S'_{stress} - S'_{rest})/S'_{rest}	
5		Time to peak systolic velocity Rest	T to S'_{rest}	ms
6		Time to peak systolic velocity Stress	T to S'_{stress}	ms
7		Time to peak systolic velocity (Stress - Rest)	T to S'_{stress} - T to S'_{rest}	ms
8		Time to peak systolic velocity [(Stress - Rest)/Rest]	(T to S'_{stress} - T to S'_{rest})/T to S'_{rest}	
9		Peak post-systolic velocity Rest	PS'_{rest}	cm/s
10		Peak post-systolic velocity Stress	PS'_{stress}	cm/s
11		Peak post-systolic velocity (Stress - Rest)	PS'_{stress} - PS'_{rest}	cm/s
12		Peak post-systolic velocity [(Stress - Rest)/Rest]	(PS'_{stress} - PS'_{rest})/PS'_{rest}	
13		Peak E‘ wave velocity Rest	E'_{rest}	cm/s
14		Peak E‘ wave velocity Stress	E'_{stress}	cm/s

15		Peak A‘ wave velocity Rest	A'_{rest}	cm/s
16		Peak A‘ wave velocity Stress	A'_{stress}	cm/s
17		E‘/A‘ ratio Rest	E'/A'_{rest}	
18		E‘/A‘ ratio Stress	E'/A'_{stress}	
19	CC-DMI: longitudinal axis	Peak systolic velocity Rest	S'_{rest}	cm/s
20		Peak systolic velocity Stress	S'_{stress}	cm/s
21		Peak systolic velocity (Stress - Rest)	$S'_{stress} - S'_{rest}$	cm/s
22		Peak systolic velocity [(Stress - Rest)/Rest]	$(S'_{stress} - S'_{rest})/S'_{rest}$	
23		Peak post-systolic velocity Rest	PS'_{rest}	cm/s
24		Peak post-systolic velocity Stress	PS'_{stress}	cm/s
25		Peak post-systolic velocity (Stress - Rest)	$PS'_{stress} - PS'_{rest}$	cm/s
26		Peak post-systolic velocity [(Stress - Rest)/Rest]	$(PS'_{stress} - PS'_{rest})/PS'_{rest}$	
27		Peak E‘ wave velocity Rest	E'_{rest}	cm/s
28		Peak E‘ wave velocity Stress	E'_{stress}	cm/s
29		Peak A‘ wave velocity Rest	A'_{rest}	cm/s
30		Peak A‘ wave velocity Stress	A'_{stress}	cm/s
31		E‘/A‘ ratio Rest	E'/A'_{rest}	

32		E‘/A‘ ratio Stress	E'/A'_{stress}	
33		Peak systolic strain Rest	SS_{rest}	%
34		Peak systolic strain Stress	SS_{stress}	%
35		Peak systolic strain (Stress - Rest)	$SS_{stress} - SS_{rest}$	%
36		Peak systolic strain [(Stress - Rest)/Rest]	$(SS_{stress} - SS_{rest})/SS_{rest}$	
37		Peak post-systolic strain Rest	PSS_{rest}	%
38		Peak post-systolic strain Stress	PSS_{stress}	%
39		Peak post-systolic strain (Stress - Rest)	$PSS_{stress} - PSS_{rest}$	%
40		Peak post-systolic strain [(Stress - Rest)/Rest]	$(PSS_{stress} - PSS_{rest})/PSS_{rest}$	
41		Time to peak post-systolic strain Rest	T to PSS_{rest}	ms
42		Time to peak post-systolic strain Stress	T to PSS_{stress}	ms
43		Time to peak post-systolic strain (Stress - Rest)	T to PSS_{stress} - T to PSS_{rest}	ms
44		Time to peak post-systolic strain [(Stress - Rest)/Rest]	(T to PSS_{stress} - T to PSS_{rest})/T to PSS_{rest}	
45		Post-systolic index Rest	PSI_{rest}	%
46		Post-systolic index Stress	PSI_{stress}	%
47		Post-systolic index (Stress - Rest)	$PSI_{stress} - PSI_{rest}$	%

48		Post-systolic index [(Stress - Rest)/Rest]	$(PSI_{stress} - PSI_{rest})/PSI_{rest}$	
49		Ratio of post-systolic index to peak systolic strain Rest	PSI_{rest}/SS_{rest}	
50		Ratio of post-systolic index to peak systolic strain Stress	PSI_{stress}/SS_{stress}	
51		Ratio of post-systolic index to peak post-systolic strain Rest	PSI_{rest}/PSS_{rest}	
52		Ratio of post-systolic index to peak post-systolic strain Stress	$PSI_{stress}/PSS_{stress}$	
53		Peak systolic strain rate Rest	SSR_{rest}	s^{-1}
54		Peak systolic strain rate Stress	SSR_{stress}	s^{-1}
55		Peak systolic strain rate (Stress - Rest)	$SSR_{stress} - SSR_{rest}$	s^{-1}
56		Peak systolic strain rate [(Stress - Rest)/Rest]	$(SSR_{stress} - SSR_{rest})/SSR_{rest}$	
57		Peak post-systolic strain rate Rest	$PSSR_{rest}$	s^{-1}
58		Peak post-systolic strain rate Stress	$PSSR_{stress}$	s^{-1}
59		Peak post-systolic strain rate (Stress - Rest)	$PSSR_{stress} - PSSR_{rest}$	s^{-1}
60		Peak post-systolic strain rate [(Stress - Rest)/Rest]	$(PSSR_{stress} - PSSR_{rest})/PSSR_{rest}$	
61	STI: longitudinal axis	Peak systolic velocity Rest	S'_{rest}	cm/s
62		Peak systolic velocity Stress	S'_{stress}	cm/s
63		Peak systolic velocity (Stress - Rest)	$S'_{stress} - S'_{rest}$	cm/s
64		Peak systolic velocity [(Stress - Rest)/Rest]	$(S'_{stress} - S'_{rest})/S'_{rest}$	

65		Peak post-systolic velocity Rest	PS'_{rest}	cm/s
66		Peak post-systolic velocity Stress	PS'_{stress}	cm/s
67		Peak post-systolic velocity (Stress - Rest)	$PS'_{stress} - PS'_{rest}$	cm/s
68		Peak post-systolic velocity [(Stress - Rest)/Rest]	$(PS'_{stress} - PS'_{rest})/PS'_{rest}$	
69		Peak E‘ wave velocity Rest	E'_{rest}	cm/s
70		Peak E‘ wave velocity Stress	E'_{stress}	cm/s
71		Peak A‘ wave velocity Rest	A'_{rest}	cm/s
72		Peak A‘ wave velocity Stress	A'_{stress}	cm/s
73		E‘/A‘ ratio Rest	E'/A'_{rest}	
74		E‘/A‘ ratio Stress	E'/A'_{stress}	
75		Peak systolic strain Rest	SS_{rest}	%
76		Peak systolic strain Stress	SS_{stress}	%
77		Peak systolic strain (Stress - Rest)	$SS_{stress} - SS_{rest}$	%
78		Peak systolic strain [(Stress - Rest)/Rest]	$(SS_{stress} - SS_{rest})/SS_{rest}$	
79		Peak post-systolic strain Rest	PSS_{rest}	%
80		Peak post-systolic strain Stress	PSS_{stress}	%
81		Peak post-systolic strain (Stress - Rest)	$PSS_{stress} - PSS_{rest}$	%

82		Peak post-systolic strain [(Stress - Rest)/Rest]	$(PSS_{stress} - PSS_{rest})/PSS_{rest}$	
45		Post-systolic index Rest	PSI_{rest}	%
46		Post-systolic index Stress	PSI_{stress}	%
47		Post-systolic index (Stress - Rest)	$PSI_{stress} - PSI_{rest}$	%
48		Post-systolic index [(Stress - Rest)/Rest]	$(PSI_{stress} - PSI_{rest})/PSI_{rest}$	
49		Ratio of post-systolic index to peak systolic strain Rest	PSI_{rest}/SS_{rest}	
50		Ratio of post-systolic index to peak systolic strain Stress	PSI_{stress}/SS_{stress}	
51		Ratio of post-systolic index to peak post-systolic strain Rest	PSI_{rest}/PSS_{rest}	
52		Ratio of post-systolic index to peak post-systolic strain Stress	$PSI_{stress}/PSS_{stress}$	
83		Peak systolic strain rate Rest	SSR_{rest}	s^{-1}
84		Peak systolic strain rate Stress	SSR_{stress}	s^{-1}
85		Peak systolic strain rate (Stress - Rest)	$SSR_{stress} - SSR_{rest}$	s^{-1}
86		Peak systolic strain rate [(Stress - Rest)/Rest]	$(SSR_{stress} - SSR_{rest})/SSR_{rest}$	
87		Peak post-systolic strain rate Rest	$PSSR_{rest}$	s^{-1}
88		Peak post-systolic strain rate Stress	$PSSR_{stress}$	s^{-1}
89		Peak post-systolic strain rate (Stress - Rest)	$PSSR_{stress} - PSSR_{rest}$	s^{-1}
90		Peak post-systolic strain rate [(Stress - Rest)/Rest]	$(PSSR_{stress} - PSSR_{rest})/PSSR_{rest}$	

91	STI: radial axis	Peak radial systolic strain rate Rest	$RSSR_{rest}$	s^{-1}
92		Peak radial systolic strain rate Stress	$RSSR_{stress}$	s^{-1}
93		Peak radial systolic strain rate (Stress - Rest)	$RSSR_{stress}$ - $RSSR_{rest}$	s^{-1}
94		Peak radial systolic strain rate [(Stress - Rest)/Rest]	($RSSR_{stress}$ - $RSSR_{rest}$)/$RSSR_{rest}$	
95		Peak radial post-systolic strain rate Rest	$RPSSR_{rest}$	s^{-1}
96		Peak radial post-systolic strain rate Stress	$RPSSR_{stress}$	s^{-1}
97		Peak radial post-systolic strain rate (Stress - Rest)	$RPSSR_{stress}$ - $RPSSR_{rest}$	s^{-1}
98		Peak radial post-systolic strain rate [(Stress - Rest)/Rest]	($RPSSR_{stress}$ - $RPSSR_{rest}$)/$RPSSR_{rest}$	
