# Supplementary material for: Quantitative approach using multiple single parameters versus visual assessment in dobutamine stress echocardiography
Source: Cardiovasc Ultrasound. 2012 Jul 30;10:31. doi: 10.1186/1476-7120-10-31 (PMC3495225; doi:10.1186/1476-7120-10-31)
Supplement: Additional file 2 — Celutkiene additional file 2 70.doc: Additional file 2. Mean values of significant predictors of stenosis in NON-STENOSED and STENOSED segments. [file 1476-7120-10-31-S2.doc]

**Additional file 2. Mean values of significant predictors of stenosis in NON-STENOSED and STENOSED segments**

| **PARAMETERS** | **NON-STENOSED** | **STENOSED** | ***P* value** |
| --- | --- | --- | --- |
| **Systolic velocities** |  |  |  |
| Peak systolic velocity Stress (PW-DMI, basal anterior segment) | 12.96 ± 3.96 | 11.96 ± 3.67 | 0.205 |
| Peak systolic velocity Stress (CC-DMI, basal anterior segment) | 8.64 ± 3.01 | 6.71 ± 2.45 | 0.002 |
| Peak systolic velocity Stress (PW-DMI, basal inferolateral segment) | 13.01 ± 4.03 | 11.11 ± 3.33 | 0.016 |
| Peak systolic velocity Stress (PW-DMI, basal inferior segment) | 13.07 ± 3.23 | 11.29 ± 3.19 | 0.013 |
| Peak systolic velocity (Stress - Rest) (PW-DMI, basal inferoseptal segment) | 5.90 ± 3.16 | 4.38 ± 3.03 | 0.020 |
| Peak systolic velocity (Stress - Rest) (PW-DMI, basal inferior segment) | 4.99 ± 2.91 | 3.63 ± 2.45 | 0.018 |
| Time to peak systolic velocity Stress (PW-DMI, basal inferoseptal segment) | 59.86 ± 16.37 | 70.00 ± 19.35 | 0.004 |
| Time to peak systolic velocity (Stress - Rest) (PW-DMI, basal inferior segment) | -60.30 ± 36.73 | -40.33 ± 32.86 | 0.022 |
| Time to peak systolic velocity (Stress - Rest) (PW-DMI, mid inferior segment) | -56.04 ± 40.39 | -39.77 ± 37.35 | 0.048 |
| Time to peak systolic velocity [(Stress - Rest)/Rest] (PW-DMI, basal inferior segment) | -0.44 ± 0.21 | -0.30 ± 0.27 | 0.009 |
| Peak post-systolic velocity [(Stress - Rest)/Rest] (CC-DMI, basal anterior segment) | 3.15 ± 10.04 | 3.97 ± 4.37 | 0.024 |
| Peak post-systolic velocity (Stress - Rest) (PW-DMI, mid anteroseptal segment) | 2.52 ± 2.68 | 3.67 ± 2.08 | 0.009 |
| Peak post-systolic velocity [(Stress - Rest)/Rest] (PW-DMI, mid anteroseptal segment) | 0.67 ± 0.86 | 1.21 ± 0.81 | 0.003 |
| **Diastolic velocities** |  |  |  |
| Peak E‘ wave velocity Rest (PW-DMI, basal inferior segment) | 8.16 ± 2.74 | 6.87 ± 2.17 | 0.018 |
| Peak E‘ wave velocity Stress (PW-DMI, basal inferior segment) | 9.18 ± 3.48 | 6.91 ± 2.57 | 0.000 |
| Peak E‘ wave velocity Stress (STI, basal inferoseptal segment) | 5.74 ± 2.35 | 4.31 ± 1.85 | 0.003 |
| Peak E‘ wave velocity Stress (PW-DMI, basal inferolateral segment) | 9.94 ± 3.55 | 8.10 ± 3.47 | 0.014 |
| Peak E‘ wave velocity Stress (CC-DMI, mid anteroseptal segment) | 3.97 ± 2.44 | 2.38 ± 1.37 | 0.001 |
| Peak A‘ wave velocity Stress (PW-DMI, basal inferoseptal segment) | 16.07 ± 4.23 | 14.03 ± 3.88 | 0.018 |
| E‘/A‘ ratio Rest (CC-DMI, basal inferoseptal segment) | 0.80 ± 0.32 | 0.64 ± 0.23 | 0.012 |
| E‘/A‘ ratio Rest (CC-DMI, mid inferolateral segment) | 0.92 ± 0.98 | 0.57 ± 0.33 | 0.017 |
| E‘/A‘ ratio Stress (STI, basal inferoseptal segment) | 0.59 ± 0.31 | 0.45 ± 0.21 | 0.023 |
| E‘/A‘ ratio Stress (PW-DMI, basal inferolateral segment) | 0.75 ± 0.38 | 0.54 ± 0.21 | 0.003 |
| **Longitudinal strain** |  |  |  |
| Peak systolic strain Rest (STI, basal inferior segment) | -19.89 ± 5.78 | -15.49 ± 8.46 | 0.010 |
| Peak systolic strain Stress (CC-DMI, mid inferior segment) | -15.20 ± 6.78 | -11.71 ± 5.36 | 0.009 |
| Peak systolic strain Stress (CC-DMI, mid inferolateral segment) | -18.05 ± 7.10 | -12.78 ± 6.07 | 0.001 |
| Post-systolic index Rest (STI, apical inferoseptal segment) | 9.08 ± 11.89 | 3.91 ± 8.57 | 0.014 |
| Post-systolic index (Stress - Rest) (STI, basal anterior segment) | 1.15 ± 10.71 | -4.81 ± 11.01 | 0.019 |
| Post-systolic index [(Stress - Rest)/Rest] (STI, mid inferior segment) | -1.42 ± 6.75 | -0.24 ± 9.95 | 0.005 |
| Ratio of post-systolic index to peak systolic strain Rest (STI, apical inferoseptal segment) | -0.33 ± 0,43 | -0.14 ± 0.35 | 0.013 |
| Ratio of post-systolic index to peak systolic strain Stress (CC-DMI, mid inferoseptal segment) | -0.07 ± 1.05 | 0.15 ± 0.51 | 0.009 |
| Ratio of post-systolic index to peak systolic strain Stress (CC-DMI, basal inferior segment) | -0.12 ± 0.46 | 0.07 ± 0.58 | 0.068 |
| Ratio of post-systolic index to peak systolic strain Stress (STI, mid anteroseptal segment) | -0.09 ± 1.01 | 0.26 ± 0.50 | 0.022 |
| **Longitudinal strain rate** |  |  |  |
| Peak systolic strain rate Stress (CC-DMI, basal anterior segment) | -2.74 ± 1.50 | -2.05 ± 0.93 | 0.002 |
| Peak systolic strain rate Stress (CC-DMI, mid inferolateral segment) | -2.24 ± 1.41 | -1.59 ± 1.09 | 0.005 |
| Peak systolic strain rate (Stress - Rest) (CC-DMI, mid inferoseptal segment) | -0.34 ± 0.99 | 0.08 ± 0.73 | 0.020 |
| Peak post-systolic strain rate Rest (STI, mid inferior segment) | -0.27 ± 0.36 | -0.50 ± 0.48 | 0.018 |
| Peak post-systolic strain rate Rest (STI, basal anterior segment) | -1.08 ± 0.78 | -0.60 ± 0.55 | 0.002 |
| Peak post-systolic strain rate (Stress - Rest) (STI, basal anterior segment) | -0.20 ± 1.06 | -0.80 ± 0.97 | 0.006 |
| **Radial strain rate (STI)** |  |  |  |
| Peak radial systolic strain rate Rest (apical inferoseptal segment) | 1.75 ± 0.80 | 1.35 ± 0.52 | 0.002 |

*PW-DMI,* pulsed wave Doppler myocardial imaging; *CC-DMI,* color coded Doppler myocardial imaging; *STI,* speckle tracking imaging
